# Rab23 Drives Cutaneous Squamous Cell Carcinoma Invasion via ERK-Mediated Autophagy Activation

**DOI:** 10.3390/cancers18213458

**Published:** 2026-10-26

**Authors:** Jiaqiang Wu, Ziye Feng, Chunyu Xie, Jingrun Yang

**Affiliations:** 1 Department of General Surgery, the First Medical Center of Chinese People’s Liberation Army General Hospital, No. 28 Fuxing Road, Hai Dian District, Beijing 100853, China; 2 Chinese Academy of Sciences Key Laboratory for Biomedical Effects of Nanomaterials and Nanosafety, Chinese Academy of Sciences Center for Excellence in Nanoscience, National Center for Nanoscience and Technology, Chinese Academy of Sciences, Beijing 100080, China

**Keywords:** Ras-associated binding protein 23, autophagy, invasion, cutaneous squamous cell carcinoma, MEK/ERK1/2

## Abstract

Background: Cutaneous squamous cell carcinoma (cSCC) is the second most prevalent non-melanoma skin cancer worldwide, with increasing incidence and considerable mortality following metastasis. Ras-associated binding protein 23 (Rab23) has been implicated in cSCC progression, but its molecular mechanisms remain incompletely understood. Methods: Rab23, Beclin1, and LC3-II protein expression was compared across human cSCC, actinic keratosis, and normal skin tissues. Rab23-dependent changes in autophagy-related markers, autophagic vacuoles, migration, invasion, and wound healing were investigated in oral SCC-derived Sa3 cells and cSCC models A431 and HSC-5. GTP dependence, responses to rapamycin and chloroquine, and AKT/mTOR and MEK/ERK1/2 signaling were assessed, including MEK1/2 inhibition with U0126. Public transcriptomic and spatial transcriptomic datasets were also analyzed. Results: Rab23 protein expression was significantly elevated in cSCC lesions compared with normal skin and actinic keratosis, accompanied by increased Beclin1 and LC3-II levels, and correlated positively with both markers. Rab23 overexpression increased Beclin1 and LC3-II expression and decreased p62 in a GTP-dependent manner. It also enhanced autophagic vacuole formation, migration, invasion, and wound healing. Rapamycin potentiated these effects, whereas chloroquine abrogated them. Rab23 activated both AKT/mTOR and MEK/ERK1/2 signaling; however, U0126 abolished Rab23-induced autophagy marker upregulation and migratory capacity. Transcriptomic and spatial transcriptomic analyses further supported the clinical relevance of the Rab23-ERK-autophagy axis in SCC aggressiveness. Conclusions: Rab23 promotes cSCC invasion, at least in part, through ERK-dependent autophagy enhancement, highlighting this signaling axis as a potential therapeutic target for cSCC.

## Simple Summary

Cutaneous squamous cell carcinoma is a common skin malignancy associated with substantial mortality after metastasis. The biological mechanisms underlying its invasive progression remain incompletely understood, limiting the development of reliable biomarkers and targeted therapies. This study examined whether Rab23, a membrane-trafficking protein, is associated with tumor invasion through regulation of autophagy. Rab23 expression was elevated in tumor specimens and correlated with autophagy-related proteins and adverse clinicopathological features. In cancer cell models, Rab23 activation was accompanied by increased autophagy, migration, and invasion. Pharmacological inhibition of autophagy or ERK signaling attenuated these effects, supporting a role for ERK-associated autophagy in Rab23-mediated invasive behavior. In addition, liposomal delivery of Rab23-silencing RNA reduced several malignant phenotypes in vitro. These findings suggest that Rab23 may contribute to cutaneous squamous cell carcinoma progression and warrant further investigation as a potential biomarker and therapeutic target.

## 1. Introduction

Cutaneous squamous cell carcinoma (cSCC) ranks as the second most common non-melanoma skin cancer worldwide, with an annual incidence increase of 3–8% among Caucasian populations [1,2]. Although most primary cSCC lesions are amenable to surgical resection and/or radiotherapy, the 5-year recurrence rate for primary tumors approaches 8%, and the mortality rate among metastatic cSCC patients escalates to approximately 40% [3,4]. These sobering statistics underscore the urgent need for a deeper mechanistic understanding of cSCC metastasis to inform the development of more effective prognostic biomarkers and targeted therapeutic strategies.

Autophagy, an evolutionarily conserved catabolic process wherein cytoplasmic constituents are sequestered within autophagic vacuoles and delivered to lysosomes for degradation, plays a context-dependent and often dichotomous role in cancer biology [5]. Emerging evidence indicates that autophagy can exert either tumor-suppressive or tumor-promoting functions depending on cell type, genetic context, and disease stage [6,7]. In cSCC, prior studies have reported that elevated expression of Beclin1 and microtubule-associated protein 1A/1B-light chain 3 (LC3), coupled with diminished p62 expression, correlates with disease progression and lymph node metastasis, portending poor clinical outcomes [8,9]. Conversely, Choi and colleagues demonstrated that autophagy activation contributes to growth inhibition and cellular senescence in metastatic cSCC cells [10]. Thus, the precise role of autophagy in cSCC development and metastasis remains an area of active investigation.

Rab23, a member of the Ras small GTPase superfamily, plays a pivotal role in intracellular membrane trafficking, tumor cell metastasis, and autophagic vacuole biogenesis [11]. Our previous work revealed that Rab23 protein levels are markedly elevated in invasive cSCC lesions relative to Bowen’s disease and actinic keratosis (AK), and that GTP-bound Rab23 potentiates cell migration and invasion, whereas Rab23 silencing exerts opposing effects [12]. In human keratinocyte HaCaT cells, Rab23 overexpression upregulates Beclin1 and LC3B-II expression, while Rab23 knockdown produces the converse phenotype [11]. Collectively, these observations led us to hypothesize that Rab23 promotes cSCC invasion through enhancement of autophagic activity.

To test this hypothesis, we systematically examined the correlation between Rab23 and autophagy marker expression in cSCC clinical specimens and employed gain- and loss-of-function approaches in Sa3, A431 and HSC-5, cSCC cells to dissect the mechanistic pathways through which Rab23 regulates autophagy and malignant progression. Our results establish Rab23 as a novel positive regulator of autophagy in cSCC that operates via ERK signaling activation, thereby promoting tumor cell invasion. These findings position Rab23 as a promising therapeutic target for mitigating cSCC invasion and metastasis.

## 2. Materials and Methods

### 2.1. Patients and Specimens

A total of 120 tissue samples collected between 2014 and 2017 were obtained from the Department of Dermatology, Chinese PLA General Hospital (Beijing, China), including 41 cSCC samples, 49 AK samples, and 30 normal skin samples. The clinical characteristics of the individuals are summarized in Table S1. Patients with histologically confirmed primary cSCC who had not received any systemic cancer therapy were included. Patients with active autoimmune disease, diabetes, inadequate renal or hepatic function, or other malignant tumors were excluded. No significant age and gender differences were observed. The pathologic diagnosis was performed by pathologists and dermatologists. The tumor stage was assessed according to the Union for International Cancer Control (UICC) version 7.0 [13]. The study was approved by the Institutional Reviewer Board of the Chinese PLA General Hospital. Written informed consent was obtained from the participants.

### 2.2. Online Data Collection

Publicly available transcriptomic datasets were obtained from the Gene Expression Omnibus (GEO) database and were classified according to their anatomical origin. GSE30784 comprises 167 oral squamous cell carcinoma (OSCC) samples, 17 oral dysplasia samples, and 45 normal oral tissue samples [14], whereas GSE9844 comprises 26 oral tongue squamous cell carcinoma (OTSCC) samples and 12 control samples [15]. Because these two datasets represent mucosal rather than cutaneous SCC, they were analyzed only as independent SCC cohorts to examine whether RAB23-associated expression patterns extend across anatomically distinct SCCs and were not used as direct validation datasets for cSCC-specific conclusions. For cSCC-specific transcriptomic analyses, GSE2503, containing normal skin, actinic keratosis (AK), and cutaneous SCC samples [16,17], and GSE86544, containing cSCC samples with different degrees of perineural invasion (PNI) [18], were used. The TCGA database contains 528 cases with HNSC. mRNA expression of Rab23 and autophagy markers were assessed using the cBio cancer genomics portal [19].

### 2.3. Immunohistochemical Staining and Participant Stratification

Paraffin-embedded tissue sections were deparaffinized, rehydrated, and subjected to hematoxylin and eosin staining for routine histopathological evaluation. Immunohistochemical staining (IHC) was performed to detect Rab23 and autophagy-associated proteins. The primary antibodies included anti-Rab23 (#11101-1-AP; 1:200; Proteintech, Rosemont, IL, USA), anti-Beclin1 (#ab114071; 1:500; Abcam, Cambridge, UK), anti-LC3II (#12741S;1:500; CST, Danvers, MA, USA), and anti-p62 (#ab280086; 1:500; Abcam). The IHC scores were calculated by multiplying the percentage of positive cells by the intensity of staining [20]. The participants were stratified into low- and high-expression groups according to the median values of the IHC scores (Table S2). Hierarchical clustering heatmap was generated using Cluster 3.0 and visualized using Java Tree View 1.1.6 software.

### 2.4. Cell Culture

Sa3 (RCB0980; RIKEN BioResource Research Center, Tsukuba, Japan), a human gingival squamous cell carcinoma cell line, was included as an SCC model based on its previous use in Rab23 studies addressing cSCC-associated invasive phenotypes. It was cultured in Dulbecco’s Modified Eagle’s Medium (DMEM) supplemented with 10% fetal bovine serum at 37 °C in a humidified atmosphere containing 5% CO_2_. A-431 and HSC-5 cells were purchased from Wuhan Procell Life Science & Technology Co., Ltd. (Wuhan, China; Cat. No. CL-0015) and Cellverse Co., Ltd. (Shanghai, China; Cat. No. iCell-h012). A-431 and HSC-5 cell complete medium provided by Procell were used according to the manufacturer’s instructions. Cells were cultured at 37 °C in a humidified incubator with 5% CO_2_. The medium was refreshed every 2–3 days, and cells were subcultured at approximately 80–90% confluence using 0.25% trypsin-EDTA.

### 2.5. Establishment of Stable Cell Lines and siRNA Transfection

SCC models stably expressing wildtype Rab23, Rab23-Q68L or Rab23-S23N mutant, small hairpin RNA against Rab23 (shRab23), or negative control shRNA (shNC) were generated as previously described [12]. Briefly, full-length human wildtype Rab23 cDNA or Rab23-Q68L or Rab23-S23N mutant was cloned at the *Nhe I/Nhe I* site of the pGC-FU-EGFP-NSC-IRES-puromycin vector. The lentiviral construct expressing shRab23 or shNC was obtained by annealing the predesigned primers and subsequent cloning into the *AgeI*-*EcoRI* sites of the GV248 lentiviral vector. Primers targeting nucleotides 668–686 of human Rab23 mRNA were 5′–CCGGTCAATCTTAGACCCAACAACTCGAGTTGTTGGGTCTAAGATTGATTTT TG–3′ (sense) and 5′–AATTCAAAAATCAATCTTAGACCCAAC AACTCG AGTTG TTGGGTCTAAGA TTGA–3′ (antisense). The lentiviral vectors were generated as previously described [12]. The stable cells were selected and maintained in DMEM containing puromycin (2 μg/mL).

The siRNAs targeting Rab23 (siRab23, sense: 5′–CAAACAAAGGACCAAGAAATT–3′, antisense: 5′–UUUCUUGGUCCUUUGUUUGTT–3′) were obtained from Genepharma (Shanghai, China). Cells were transfected with siRNAs using Lipofectamine 2000 (Invitrogen, Carsland, CA, USA) following the manufacturer’s instruction.

### 2.6. Cell Migration and Invasion Assays

Cell migration and invasion assays were performed at 48 h after transfection. In the migration assay, cells were resuspended in 200 µL serum-free DMEM and seeded in the upper chamber inserted in a well of a 24-well plate. In the invasion assay, the resuspended cells were seeded in a Matrigel-coated Transwell chamber (Corning, Corning, NY, USA). The lower chamber was filled with DMEM containing 10% FBS. Cells were allowed to migrate or invade for 20 h. The migrating or invading cells on the lower surface of the chamber were fixed with 100% methanol and stained with 1% crystal violet. Cells were counted in 5 randomly selected fields. Images were acquired under an inverted light microscope at 200× magnification.

In wound-healing assay, cells were seeded in a cell culture plate and serum-starved overnight after reaching 90% confluence. A scratch was made using a 200 μL pipette tip, followed by phosphate-buffered solution (PBS) rinses. Cells were allowed to migrate for 24 h in serum-free medium. Images of the wound were acquired using an inverted microscope (Nikon, Tokyo, Japan). The migration area was calculated using Image J (version 1.53k, National Institutes of Health (NIH), Bethesda, MD, USA).

### 2.7. Monodansylcadaverine Staining and Confocal Microscopy

MDC staining was used as a complementary acidotropic-dye assay to visualize acidic vesicular compartments [21]. Briefly, cells were incubated with 0.05 mM MDC for 15 min at 37 °C, washed with PBS, and observed under fluorescence microscope. Colocalization of autophagic vacuoles and Rab23 (represented by GFP fluorescence) was observed under an inverted confocal microscope (Leica Microsystems, Wetzlar, German). The colocalization rate was calculated as the percentage of the colocalized pixel intensities to the total pixel intensities.

### 2.8. Western Blot Analysis

Proteins were isolated from Sa3 cells using RIPA buffer (Solarbio, Beijing, China) and quantified using BCA method. Proteins (20 µg) were separated using an SDS-PAGE gel and then transferred to PVDF membranes. The membranes were incubated with anti-Rab23 (#11101-1-AP; 1:500; Proteintech), anti-Beclin1 (#ab114071; 1:500; Abcam), anti-LC3-II (#12741S; 1:1000; CST), anti-p62 (#ab280086; 1:1000; Abcam), anti-AKT (#4685S; 1:1000; CST); anti-p-AKTS473 (#4060S; 1:2000; CST), anti-mTOR (#2983S; 1:1000; CST), anti-p-mTOR^S2448^ (#5536S; 1:1000; CST), anti-MEK1/2 (#8727S; 1:1000; CST), anti-p-MEK^S217/221^ (#9154S; 1:1000; CST), anti-ERK1/2 (#4695S; 1:1000; CST), anti-p-ERK1/2^T202/204^ (#4370S; 1:1000; CST), anti-β-actin (#CW0096M; 1:5000; Cwbiotech, Taizhou, China), or anti-GAPDH (#CW0100; 1:5000, Cwbiotech) antibody at 4 °C overnight. After incubation with HRP-coupled IgG (1:2000; CST) at 37 °C for 1 h, antibody-bound proteins were visualized using ECL (Pierce, Rockford, IL, USA) and a Las-3000 luminescent image analyzer (Fujifilm, New York, NY, USA).

### 2.9. Public Transcriptomic and Spatial Transcriptomic Analyses

This analysis was based on transcriptomic data from 253 SCC tumor samples obtained from The Cancer Genome Atlas (TCGA) database. The relevant pathway gene sets were retrieved from the GSEA Molecular Signatures Database (https://www.gsea-msigdb.org/gsea/msigdb/index.jsp, accessed on 12 May 2026). RAB23 was used as the core gene, and Pearson correlation coefficients were calculated to evaluate the strength of association between RAB23 and related genes or gene sets. Multiple testing correction was performed using the Benjamini–Hochberg method to determine whether the target gene sets showed coordinated enrichment within the RAB23-associated gene network. At the single-sample level, combined z-score activity scores were calculated based on the same gene sets, and Pearson correlation scatter plots were generated to visualize the associations between RAB23 expression and each pathway activity score. In addition, samples were divided into high- and low-expression groups according to the median expression level of RAB23, and beeswarm plots were used to compare differences in gene set activity scores between the two groups.

Spatial transcriptomic data were obtained from the 10x Visium spatial transcriptomic Seurat object of the GSE144239 GSM4565826-P6-rep2 section. UCell was then applied to calculate gene set activity scores for each spot. This algorithm is based on the Mann–Whitney U statistic and evaluates whether the expression ranks of the target gene set in each spot are significantly higher than expected by chance. After scoring, multidimensional analyses were further performed. For spatial visualization, the Seurat SpatialFeaturePlot function was used to map gene set activity scores and individual gene expression levels onto the spatial coordinates of the tissue section. For boundary-region differential analysis, sections containing Cottrazm-BoundaryDefine boundary annotations were analyzed by classifying spots into Mal, Bdy, and nMal regions according to the Area Bdy annotation. Differences in gene set activity scores and individual gene expression levels among these regions were then assessed using the Wilcoxon rank-sum test.

### 2.10. Preparation and Characterization of siRab23-Loaded Liposomes

Liposomes/siRNAs were constructed following a previously reported thin-film hydration method. Briefly, DOTAP, DSPC, cholesterol, and DSPE-PEG2000 were dissolved in ethanol at a molar ratio of 20:30:18:1.5. The lipid mixture was then combined with Rab23 siRNA in acetate buffer at a siRNA-to-lipid weight ratio of 1:15, with a flow rate of 2 mL min^−1^ and a pH of 4.5. The resulting liposomes/siRNAs were dialyzed against PBS buffer for 24 h to remove ethanol and adjust the pH to neutral. Particle size and morphology were characterized by dynamic light scattering (DLS) using a Zetasizer system v7.13 and by transmission electron microscopy (TEM), respectively.

### 2.11. Statistical Analysis

Data are expressed as mean ± standard deviation (SD) unless otherwise specified. Intergroup differences in Rab23 and autophagy protein expression among normal skin, AK, and cSCC groups were assessed using the Kruskal–Wallis test followed by Dunn’s post hoc test. Correlations between Rab23 and autophagy markers were evaluated using Spearman’s rank correlation test. Associations between protein expression and clinicopathological parameters were analyzed using the chi-square or Fisher’s exact test. For in vitro experiments, two-group comparisons were conducted using Student’s *t*-test, and multi-group comparisons were performed using one-way or two-way ANOVA with appropriate post hoc tests. All statistical analyses were carried out using SPSS version 22.0 (IBM) and GraphPad Prism 6.0 (GraphPad Software). Statistical significance was defined as *p* < 0.05.

## 3. Results

### 3.1. Rab23 and Autophagy-Related Markers Show Altered Expression Patterns in cSCC Lesions

**Figure 1:**
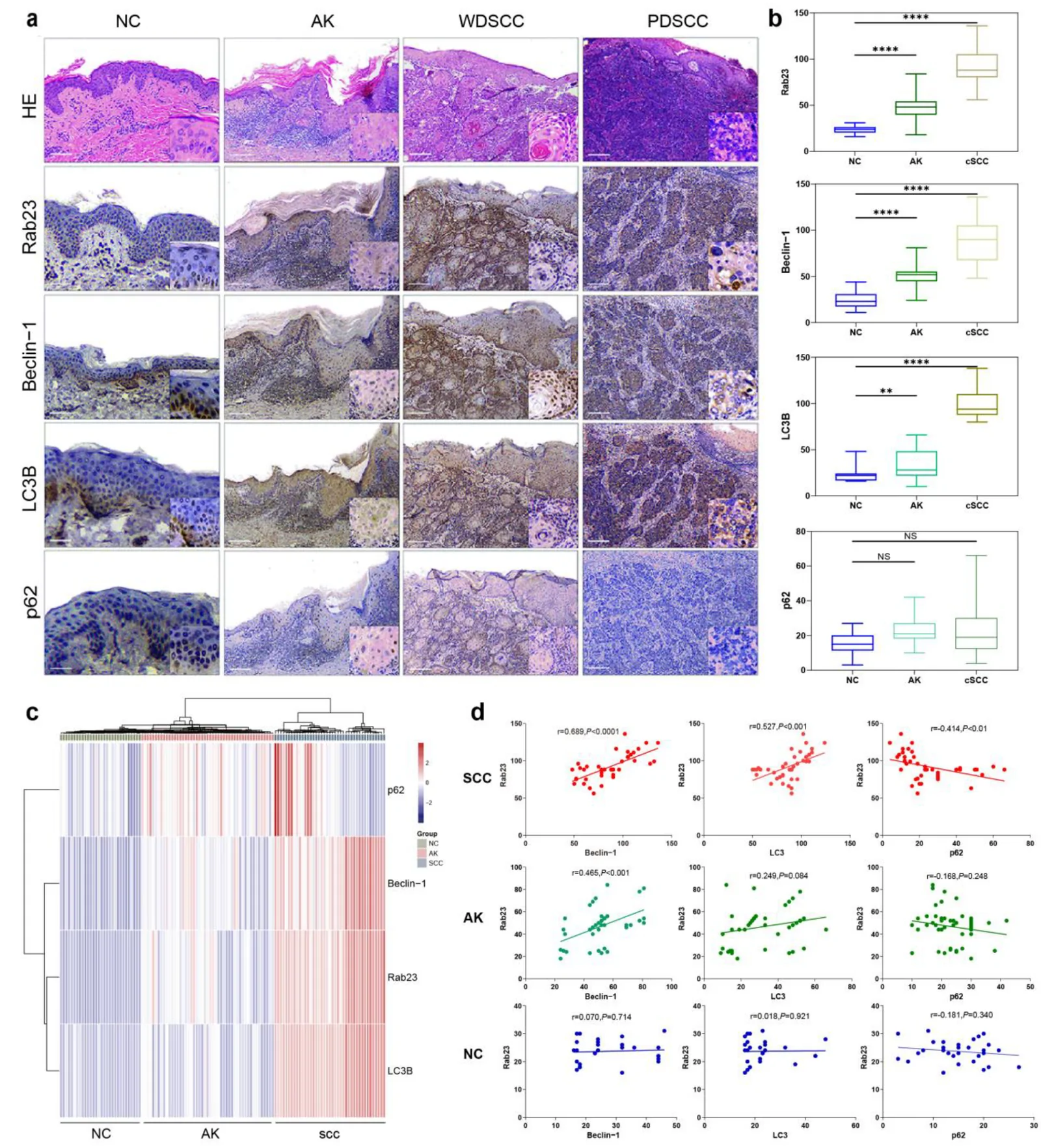
Expression patterns of autophagy-related proteins during the progression from normal skin to cutaneous squamous cell carcinoma (cSCC). (**a**) Representative hematoxylin and eosin (HE) staining and immunohistochemical (IHC) staining of Rab23, Beclin-1, LC3B, and p62 in normal control (NC), actinic keratosis (AK), well-differentiated SCC (WDSCC), and poorly differentiated SCC (PDSCC) tissues. (**b**) Quantitative analysis of Rab23, Beclin-1, LC3B, and p62 expression levels among NC, AK, and cSCC groups. Data are presented as box plots; statistical significance is indicated (** *p* < 0.01, **** *p* < 0.0001). (**c**) Heatmap showing the expression profiles of autophagy-related proteins across samples, with hierarchical clustering distinguishing NC, AK, and SCC groups. (**d**) Correlation analyses between Rab23 and Beclin-1, LC3B, and p62 in NC, AK, and SCC tissues. Spearman correlation coefficients (r) and *p*-values are indicated. HE: hematoxylin and eosin; Rab23: Ras-associated binding protein 23; LC3: microtubule-associated protein 1A/1B-light chain 3; NC: normal control; AK: actinic keratosis; cSCC: cutaneous squamous cell carcinoma; WDSCC: well-differentiated squamous cell carcinoma; PDSCC: poorly differentiated squamous cell carcinoma. Scale bars = 50 μm.

To investigate the potential involvement of Rab23 and autophagy in cSCC pathogenesis, we examined their protein expression in clinical specimens. IHC analysis revealed increased Rab23, Beclin1, and LC3-II expression in cSCC lesions, whereas p62 showed a heterogeneous expression pattern without a significant overall difference among NC, AK, and cSCC groups (Figure 1a,b). Notably, Rab23, Beclin1, and LC3-II staining intensities were further increased in moderately/poorly differentiated SCC (PDSCC) relative to well-differentiated SCC (WDSCC) (Figure 1a,b). Hierarchical clustering was conducted to assess overall survival and distinctly separated normal skin, AK, WDSCC, and PDSCC groups based on their expression profiles (Figure 1c). Correlation analyses revealed that Rab23 expression positively correlated with Beclin1 and LC3-II but negatively correlated with p62 in cSCC specimens (Figure 1d). No significant correlations were observed in normal skin or AK tissues, with the exception of a positive correlation between Rab23 and Beclin1 in AK lesions (Figure 1d). Furthermore, Rab23/Beclin1/LC3B/p62 expression levels were significantly associated with histological grade, and Beclin1/LC3B expression correlated positively with UICC stage and tumor size (Table S1). These findings suggest coordinated changes in Rab23 and autophagy-related markers during cSCC progression. Analysis of publicly available transcriptomic datasets corroborated the clinical relevance of Rab23 upregulation. RAB23 mRNA levels were significantly elevated in cSCC versus normal tissues across the GSE30784, GSE9844, and GSE2503 cohorts. In the GSE86544 dataset, RAB23 expression was highest in cSCC samples with clinically confirmed PNI compared with those with incidental or no PNI (Supplementary Figure S1A), implicating Rab23 in cSCC invasion. Similarly, TCGA analysis demonstrated elevated RAB23, LC3-II, and BECN1 mRNA in SCC versus non-cancerous tissues (Supplementary Figure S1B). In the TCGA-HNSC cohort, concurrent high expression of RAB23 and selected autophagy-related genes, including BECN1, MAP1LC3B, and SQSTM1, was associated with cSCC progression and clinicopathological features (Supplementary Figure S1C). This transcript-level association was considered complementary evidence across SCC and was not interpreted as a direct measure of autophagic activity in cSCC. Collectively, these data suggest that Rab23 upregulation, in concert with autophagy marker activation, is associated with cSCC progression.

### 3.2. Rab23 Activation Promotes Autophagy and Enhances Malignant Phenotypes in cSCC Cells

**Figure 2:**
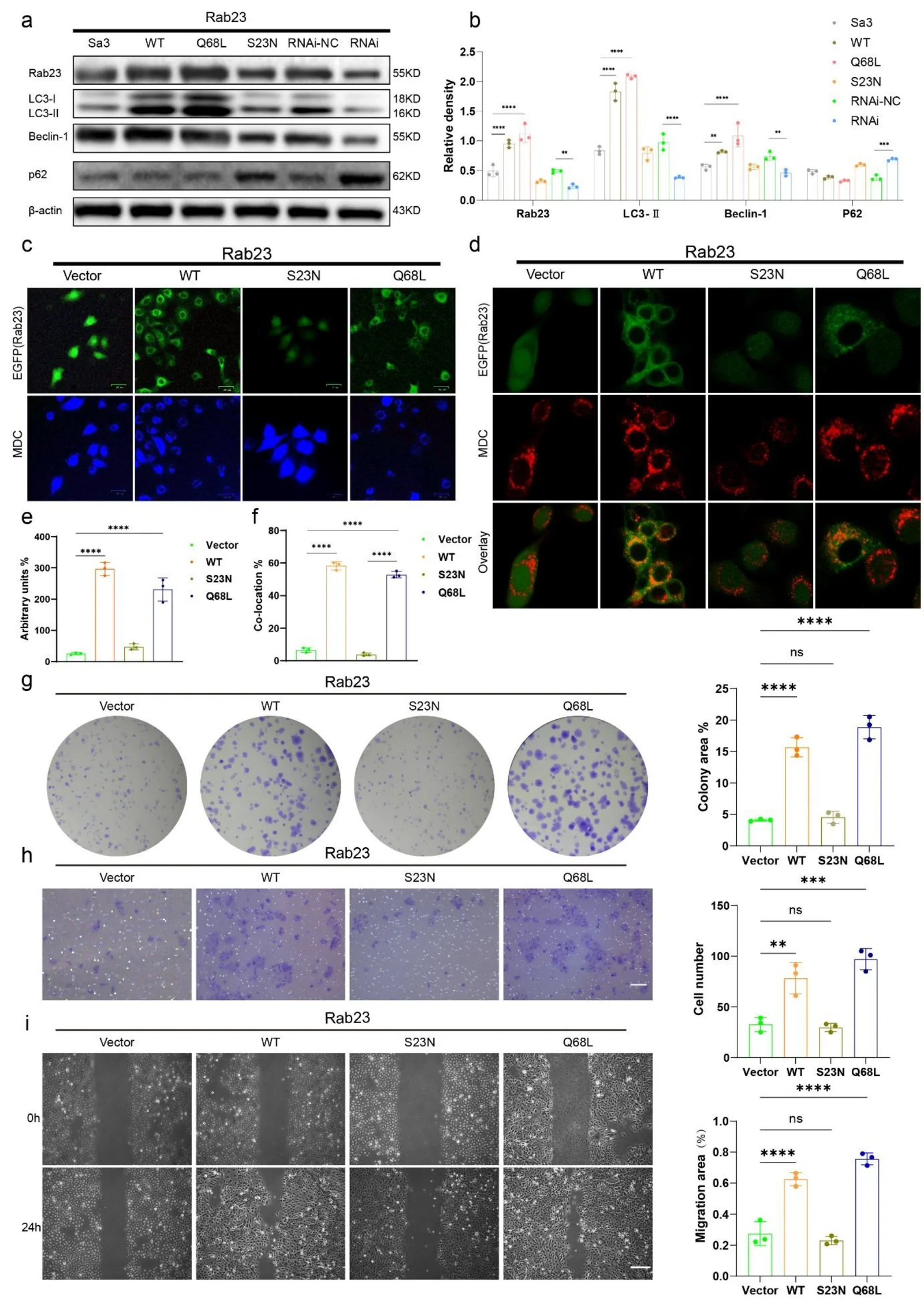
Activation of Rab23 promotes autophagy and enhances malignant phenotypes in cSCC cells. (**a**) Western blot analysis of Rab23, LC3, Beclin-1, and p62 in Sa3 cells transfected with vector control, wild-type Rab23 (WT), constitutively active mutant (Q68L), or dominant-negative mutant (S23N) or subjected to Rab23 knockdown (RNAi). β-actin was used as a loading control. (**b**) Quantitative analysis of protein expression levels shown in (**a**). Data are presented as relative density. (**c**) Fluorescence images showing EGFP-tagged Rab23 expression and MDC staining in cells transfected with vector, WT, S23N, or Q68L constructs. (**d**) Representative images of co-localization between EGFP-Rab23 (green) and MDC-labeled autophagic vacuoles (red). Merged images indicate co-localization. (**e**) Quantification of MDC fluorescence intensity (*n* = 3). (**f**) Quantification of co-localization efficiency between Rab23 and autophagic vacuoles (*n* = 3). (**g**) Colony formation assay showing proliferative capacity of cells expressing vector, WT, S23N, or Q68L Rab23. (**h**) Transwell invasion assay assessing invasive ability. (**i**) Wound-healing assay evaluating cell migration at 0 h and 24 h (*n* = 3). Statistical significance is indicated as ** *p* < 0.01, *** *p* < 0.001, **** *p* < 0.0001; ns, not significant. Scale bars = 50 μm.

To determine whether Rab23 directly regulates autophagy in cSCC cells, we examined autophagy marker expression in SCC cells with differential Rab23 activity. Western blot analysis revealed that in Sa3 cells, overexpression of wild-type Rab23 or the constitutively active GTP-bound mutant (Q68L) significantly upregulated Beclin1 and LC3-II levels while downregulating p62, whereas that of the dominant-negative GDP-bound mutant (S23N) or shRab23 knockdown produced opposite effects (Figure 2a,b). These data indicate that Rab23 induces GTP-dependent changes in autophagy-related markers. MDC staining showed an increased abundance of MDC-positive acidic vesicular compartments in cells expressing WT or Q68L Rab23 compared with vector or S23N cells (Figure 2c,e). EGFP-Rab23 fluorescence also showed increased colocalization with MDC-positive compartments in WT and Q68L cells (Figure 2d,f). Because MDC is an acidotropic dye that is not specific for autophagosomes, these observations were interpreted as complementary evidence of Rab23-associated changes in acidic vesicular compartments rather than as direct evidence of autophagosome formation. Together with the corresponding changes in Beclin1, LC3-II, and p62, these findings are consistent with an association between Rab23 activity and autophagy-related cellular processes. Functional assays demonstrated that Rab23 activation enhanced the malignant behavior of Sa3 cells. Colony formation assays showed significantly increased clonal growth in WT and Q68L groups compared with vector controls (Figure 2g). Transwell invasion and wound-healing assays revealed that WT and Q68L Rab23 overexpression substantially increased cell migration and invasion, whereas S23N expression had no significant effect (Figure 2h,i). Consistently, in HSC-5 and A431 cells, Rab23 WT and Q68L markedly enhanced Transwell migration and wound closure compared with vector controls, while S23N showed limited migratory activity (Supplementary Figure S2). Together, these findings indicate that Rab23 activation coordinately promotes autophagy and enhances the proliferative, migratory, and invasive capacities of cSCC cells.

### 3.3. Rab23 Promotes cSCC Cell Migration and Invasion Through Autophagy-Dependent Mechanisms

**Figure 3:**
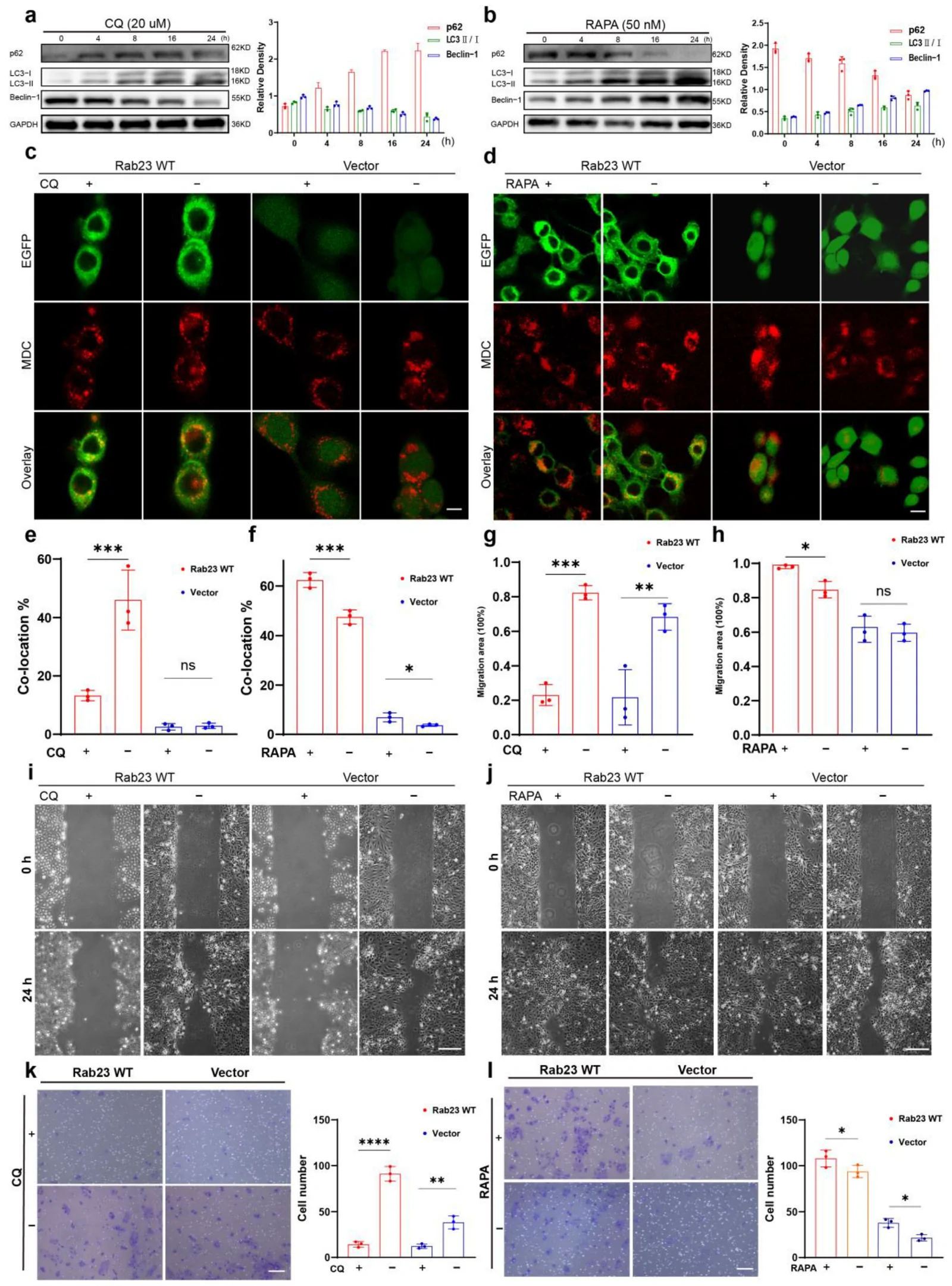
Rab23 promotes malignant behaviors of cSCC cells through autophagy-dependent mechanisms. (**a**) Western blot analysis of autophagy-related proteins (p62, LC3, and Beclin-1) in Sa3 cells treated with chloroquine (CQ, 20 μM) for the indicated time points. (**b**) Western blot analysis of autophagy markers in cells treated with rapamycin (RAPA, 50 nM). Quantification of protein levels is shown on the right. (**c**) Fluorescence images showing EGFP-Rab23 expression and MDC staining in Rab23 WT or vector-transfected cells with or without CQ treatment. (**d**) Fluorescence images of EGFP-Rab23 and MDC staining in cells treated with or without RAPA. (**e**,**f**) Quantification of colocalization between EGFP-Rab23 and MDC-positive acidic vesicular compartments under CQ or RAPA treatment. (**g**–**j**) Wound-healing assays showing migration ability of Rab23 WT or vector cells in the presence or absence of CQ (**g**,**i**) or RAPA (**h**,**j**). (**k**,**l**) Transwell invasion assays evaluating invasive capacity under CQ (**k**) or RAPA (**l**) treatment. Quantitative analyses are shown on the right (*n* = 3). Statistical significance is indicated as * *p* < 0.05, ** *p* < 0.01, *** *p* < 0.001, **** *p* < 0.0001; ns, not significant.

To establish whether autophagy is required for Rab23-mediated enhancement of malignant phenotypes, we first examined the effects of pharmacological modulation of autophagy on Rab23-overexpressing cells. Chloroquine (CQ) treatment, which inhibits autophagic flux, reduced Beclin1 and LC3-II levels while increasing p62 accumulation over time (Figure 3a). Conversely, rapamycin (RAPA) treatment, which activates autophagy, increased Beclin1 and LC3-II expression and reduced p62 levels (Figure 3b). MDC staining demonstrated that Rab23 WT cells exhibited more abundant autophagic vacuoles than vector controls, with prominent Rab23–autophagic vacuole co-localization. CQ treatment significantly attenuated this co-localization (Figure 3c,e), whereas RAPA treatment further enhanced it (Figure 3d,f). These results indicate that pharmacological modulation of autophagy-related processes affects Rab23-associated vesicular changes. Functional assays revealed that CQ treatment markedly suppressed wound closure in Rab23 WT cells, with the migratory advantage of Rab23 overexpression being significantly diminished (Figure 3g,i). In contrast, RAPA treatment maintained or further enhanced the migratory capacity of Rab23 WT cells relative to vector controls (Figure 3h,j). Transwell invasion assays yielded concordant results: CQ substantially reduced the invasive capacity of Rab23 WT cells (Figure 3k), while RAPA further augmented it (Figure 3l). Consistently, in HSC-5 and A431 cells, CQ attenuated Rab23 WT-induced wound closure and Transwell migration, whereas RAPA maintained or enhanced these effects (Supplementary Figure S3). Collectively, these findings support the involvement of autophagy-related processes in Rab23-associated cell migration and invasion.

### 3.4. Rab23 Drives Autophagy and cSCC Cell Invasion via ERK Pathway Activation

**Figure 4:**
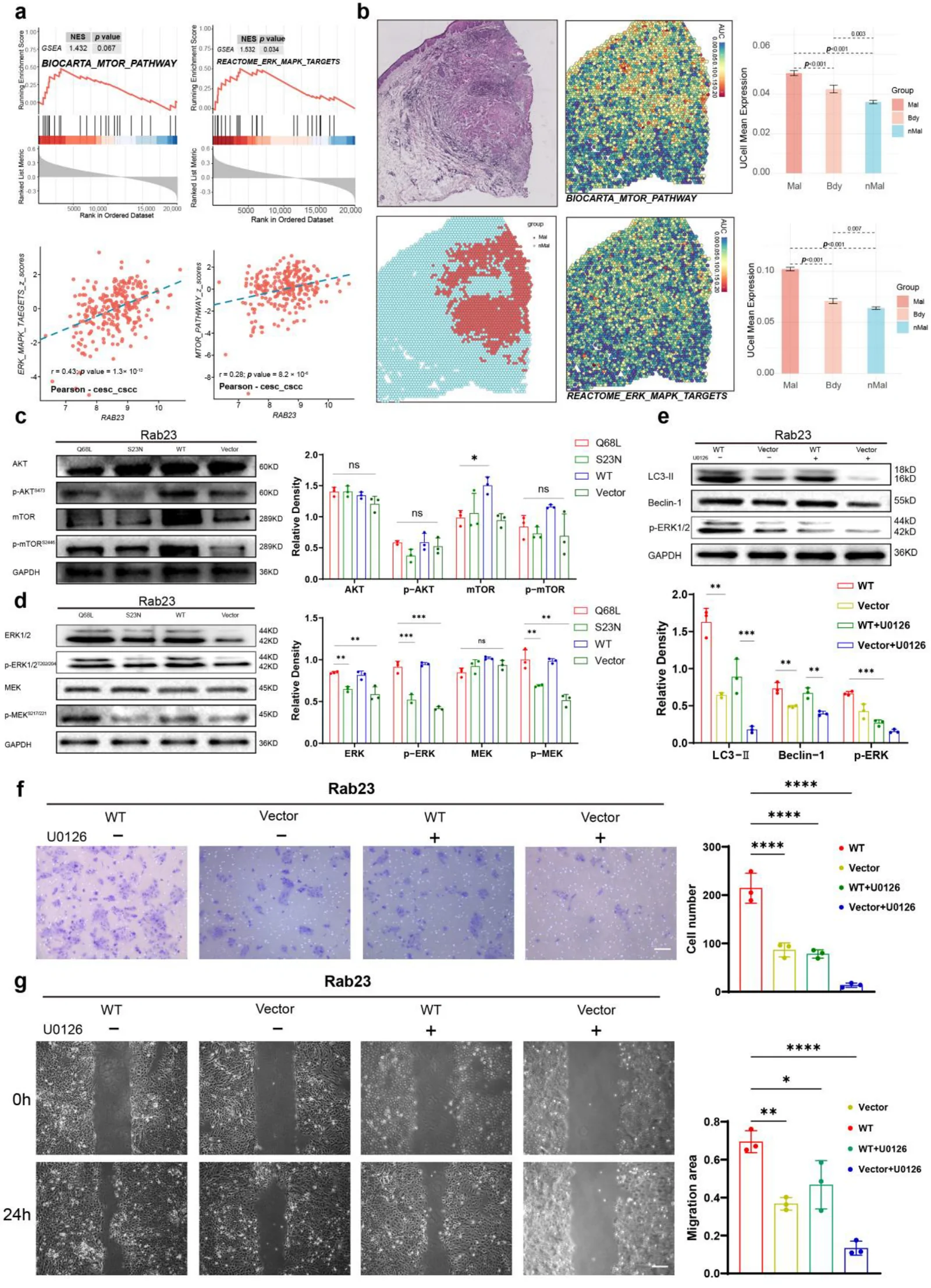
Rab23 promotes autophagy and malignant phenotypes via activation of the ERK/MAPK pathway. (**a**) Gene set enrichment analysis (GSEA) showing enrichment of mTOR signaling and ERK/MAPK target pathways in Rab23-associated gene expression profiles. Correlation analysis between Rab23 expression and pathway scores is shown below. (**b**) Spatial transcriptomic analysis showing distribution of pathway activities (mTOR and ERK/MAPK) across tissue sections, with corresponding quantification of pathway expression in malignant (Mal), boundary (Bdy), and non-malignant (nMal) regions. (**c**) Western blot analysis of AKT/mTOR signaling components (AKT, p-AKT, mTOR, p-mTOR) in cells expressing Rab23 WT, Q68L, S23N, or vector control, with quantification on the right. (**d**) Western blot analysis of ERK/MAPK signaling (MEK, p-MEK, ERK, p-ERK) under different Rab23 conditions. (**e**) Western blot analysis of p-ERK, Beclin-1, and LC3-II in the presence or absence of the MEK1/2 inhibitor U0126. (**f**) Transwell invasion assay assessing the effect of U0126 on Rab23-induced invasion. (**g**) Wound-healing assay showing migration ability under ERK inhibition. Quantitative analyses are shown on the right. Statistical significance was determined using one-way ANOVA followed by appropriate post hoc tests. ns, not significant; * *p* < 0.05, ** *p* < 0.01; *** *p* < 0.001, **** *p* < 0.0001 (*n* = 3).

To elucidate the molecular signaling through which Rab23 regulates autophagy, we performed transcriptomic and spatial transcriptomic analyses. GSEA revealed significant positive enrichment of BIOCARTA_MTOR_PATHWAY and REACTOME_ERK_ MAPK_TARGETS in RAB23-high tumors, with RAB23 expression positively correlating with both mTOR and ERK/MAPK pathway activity scores (Figure 4a). Spatial transcriptomic analysis demonstrated that both mTOR and ERK/MAPK pathway signatures were predominantly enriched in malignant regions, with significantly higher activity scores in Mal versus Bdy and nMal regions (Figure 4b), implicating these pathways in cSCC progression. Western blot analysis of key signaling nodes in Sa3 cells revealed that both WT and Q68L Rab23 upregulated phosphorylation of AKT^S473, mTOR^S2448, MEK^S217/221, and ERK1/2^T202/204, whereas S23N suppressed these phosphorylation events (Figure 4c,d). Rab23 WT and Q68L increased phosphorylation of components of both the AKT/mTOR and MEK/ERK pathways, suggesting that Rab23 may influence multiple signaling pathways associated with malignant progression. Importantly, Rab23-induced ERK activation coincided with elevated Beclin1 expression and increased LC3-II/LC3-I ratio (Figure 4e).

To examine the functional involvement of MEK/ERK signaling in Rab23-associated autophagy-related changes, Rab23 WT cells were treated with the MEK1/2 inhibitor U0126. U0126 treatment effectively suppressed p-ERK levels and concomitantly reduced Beclin1 expression and LC3-II/LC3-I ratio (Figure 4e), supporting the involvement of MEK/ERK signaling in Rab23-associated autophagy-related changes. Functionally, U0126 significantly attenuated the pro-invasive and pro-migratory effects of Rab23 overexpression, as demonstrated by Transwell invasion and wound-healing assays (Figure 4f,g). Consistently, in HSC-5 and A431 cells, U0126 markedly suppressed Rab23 WT-induced wound closure and Transwell migration (Supplementary Figure S4). These findings support a functional contribution of MEK/ERK signaling to Rab23-associated autophagy-related changes and migratory/invasive phenotypes. However, the contribution of AKT/mTOR signaling was not functionally examined and therefore remains to be determined.

### 3.5. siRab23-Loaded Liposomes Suppress Rab23 Expression and cSCC Malignant Phenotypes

**Figure 5:**
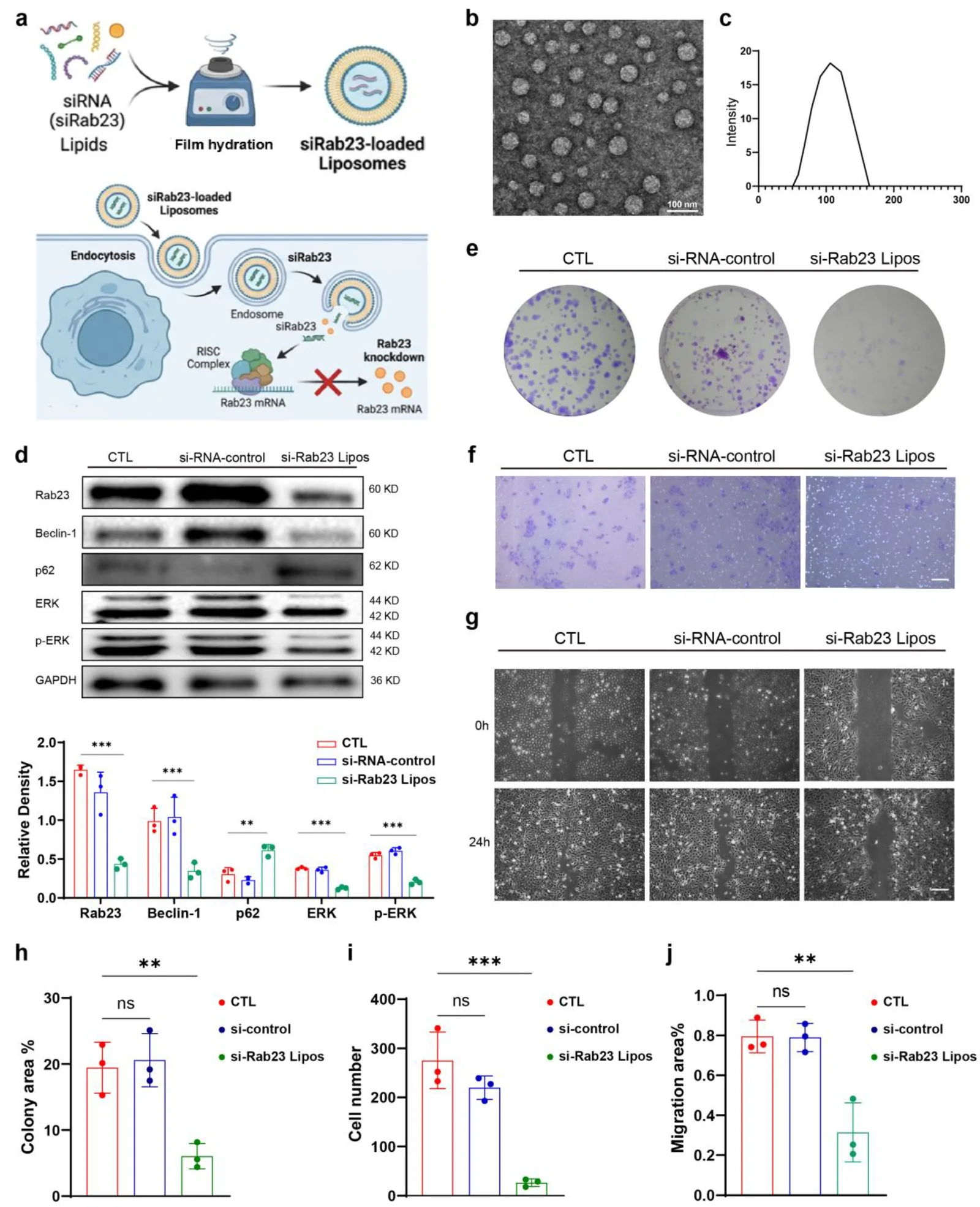
Preparation and biological effects of siRab23-loaded liposomes on tumor cells. (**a**) Schematic illustration of the preparation and intracellular delivery mechanism of siRab23-loaded liposomes. (**b**,**c**) TEM image and particle size distribution of siRab23-loaded liposomes. (**d**) Western blot analysis of Rab23, autophagy-related proteins, and ERK/p-ERK expression. (**e**,**h**) Colony formation assay and quantitative analysis. (**f**,**i**) Transwell invasion assay and quantitative analysis. (**g**,**j**) Wound-healing assay and quantitative analysis. Data are presented as mean ± SD (*n* = 3 independent experiments). Statistical significance was determined using one-way ANOVA followed by appropriate post hoc tests. ns, not significant; ** *p* < 0.01; *** *p* < 0.001 (*n* = 3).

To explore the therapeutic potential of Rab23 targeting, we developed siRab23-loaded liposomes using the thin-film hydration method (Figure 5a). TEM revealed uniform spherical nanoparticles (Figure 5b) with a mean diameter of approximately 100 nm (Figure 5c), confirming favorable nanocarrier characteristics. Western blot analysis demonstrated that siRab23 liposomes effectively suppressed Rab23 protein expression, accompanied by reduced Beclin1 and p-ERK levels and accumulation of p62 (Figure 5d). These molecular changes translated into functional consequences: siRab23 liposome treatment significantly reduced colony formation, invasive capacity, and wound-healing ability of Sa3 cells (Figure 5e–g). Notably, no significant differences were observed between siRNA-control and untreated groups, confirming that the observed effects were specifically attributable to Rab23 silencing. These results validate Rab23 as a potential therapeutic target and demonstrate the feasibility of siRNA-based Rab23 delivery for cSCC intervention.

## 4. Discussion

In this study, we have identified Rab23 as a novel and critical regulator of autophagy in cSCC, and we have elucidated a mechanistic axis through which Rab23 potentiates tumor invasion. Our findings establish that Rab23 is overexpressed in cSCC lesions and is associated with autophagy-related marker expression and adverse clinicopathological features. Mechanistically, Rab23 drives autophagy in a GTP-dependent manner through preferential activation of ERK signaling, and this autophagic program is essential for Rab23-mediated promotion of cSCC cell migration and invasion. These observations position Rab23 as a promising therapeutic target for cSCC and provide a molecular framework for understanding the interplay between membrane trafficking, autophagy, and cancer progression.

Rab proteins are increasingly recognized as integral components of the autophagic machinery, participating in distinct stages from autophagosome initiation to lysosomal fusion [22,23,24]. Our observation that Rab23 promotes autophagy in a GTP-dependent manner is consistent with the established roles of other Rab family members such as Rab1, Rab7, and Rab33 in autophagic pathway regulation. Furthermore, the co-localization of Rab23 with autophagic vacuoles at the plasma membrane and cytoplasmic compartments suggests that Rab23 may function at the nexus of membrane trafficking and autophagosome biogenesis. However, the precise molecular interactions through which Rab23 interfaces with the core autophagic machinery—particularly the Beclin1–PI3KC3 complex—warrant further investigation.

The functional significance of the Rab23-autophagy axis in cSCC invasion is underscored by our pharmacological and genetic interventions. Chloroquine treatment abrogated Rab23-induced migration and invasion, whereas rapamycin potentiated these effects. These findings align with emerging evidence that autophagy can facilitate tumor cell invasion and metastasis in specific cancer types [7,25]. However, the pro- vs. anti-metastatic roles of autophagy remain context-dependent. Our data firmly place cSCC within the paradigm wherein autophagy supports invasive progression, highlighting the need for careful contextualization when considering autophagy-targeted therapeutic strategies [26,27].

Mechanistically, Rab23 overexpression was associated with activation of both AKT/mTOR and MEK/ERK signaling. Inhibition of MEK1/2 with U0126 attenuated Rab23-associated changes in autophagy-related markers as well as migratory and invasive phenotypes, supporting a functional contribution of MEK/ERK signaling [28,29,30]. However, because the AKT/mTOR pathway was not subjected to a comparable pathway-specific functional analysis, the relative contributions of these two signaling pathways cannot be determined from the present data. Moreover, the biological significance of Rab23-associated mTOR activation in the context of autophagy regulation remains unresolved. Future studies employing pathway-specific perturbation will be required to clarify the contribution of AKT/mTOR signaling and its interaction with MEK/ERK signaling [31,32,33,34,35].

The development of siRab23-loaded liposomes in this study represents a proof-of-concept for the therapeutic targeting of Rab23. The effective knockdown of Rab23 and consequent suppression of autophagy markers, ERK activation, and malignant phenotypes validate Rab23 as a pharmacologically actionable target. However, several challenges remain. First, although the ERK pathway appears to be the predominant effector, a definitive role for AKT/mTOR signaling in Rab23-mediated autophagy cannot be entirely excluded and merits further investigation using pathway-specific inhibitors. Second, the precise molecular mechanism through which Rab23 activates ERK signaling—whether through direct protein–protein interactions, modulation of upstream receptors, or regulation of membrane trafficking of signaling components—remains to be elucidated. Third, in vivo validation using orthotopic or genetically engineered mouse models of cSCC is essential to confirm the pathophysiological relevance of the Rab23-ERK-autophagy axis and to evaluate the therapeutic efficacy of Rab23-targeted interventions in a physiologically relevant context. Our study indicates that Rab23 is a novel regulator of autophagy in cSCC cells. Rab23 enhances autophagy by activating ERK signaling in cSCC cells and thereby promotes cSCC cell migration and invasion. Targeting Rab23 and ERK signaling is a promising therapeutic strategy against cSCC invasion.

Several limitations of the present study should be considered. First, normal skin was used as the non-neoplastic reference because matched perilesional skin samples were unavailable. Given that perilesional skin shares ultraviolet and environmental exposure with the tumor-bearing field and may already exhibit molecular alterations associated with field cancerization, comparisons between cSCC and normal skin may not completely distinguish tumor-specific changes from those related to chronic exposure. Future studies comparing matched cSCC, perilesional skin, and normal skin will help determine when Rab23 upregulation and autophagy-associated alterations emerge during cSCC development. Second, part of the mechanistic investigation was performed in Sa3 cells, which originate from oral rather than cutaneous squamous cell carcinoma. Although Rab23-associated migratory and invasive phenotypes and their responses to autophagy or ERK modulation were supported in additional SCC models, biological differences between mucosal and cutaneous SCC should be taken into account. Validation in additional well-characterized cSCC models is therefore needed to confirm the generalizability and cutaneous specificity of the proposed mechanism. Third, MDC staining was used as supportive evidence of changes in acidic vesicular compartments rather than as a specific measure of autophagosomes or autophagic flux. Although the concomitant changes in Beclin1, LC3-II, and p62, together with the responses to pharmacological modulation, support the involvement of autophagy-associated processes, future studies should directly assess autophagic flux using tandem fluorescent LC3 reporters and lysosomal turnover assays.

In summary, our study identifies Rab23 as a regulator of autophagy-associated processes in cSCC and supports a model in which Rab23 promotes ERK activation, enhances autophagy, and facilitates tumor cell migration and invasion. These findings provide a mechanistic rationale for targeting the Rab23–ERK axis as a potential therapeutic strategy to limit cSCC invasiveness, while further mechanistic, in vivo, and translational validation will be required before clinical application.

## 5. Conclusions

Collectively, our findings support a role for Rab23 in promoting SCC invasive phenotypes and identify MEK/ERK signaling as an important functional contributor to Rab23-associated autophagy-related changes and migration/invasion. The clinical correlation of Rab23 with autophagy marker expression, its association with clinicopathological features and the functional findings support further investigation of Rab23 as a potential biomarker and therapeutic target. Future studies aimed at elucidating the upstream regulators and downstream effectors of Rab23 in the autophagic pathway will further inform the development of targeted strategies to mitigate cSCC progression.

## Data Availability

Data were generated by the authors and are available on request.

## Supplementary Materials

The following supporting information can be downloaded at: https://www.mdpi.com/article/10.3390/cancers18213458/s1, Figure S1. Rab23 mRNA was overexpressed in patients with cSCC from GEO and TCGA datasets and correlated with short overall survival of patients. Figure S2. Rab23 enhances the migration of HSC-5 and A431 cells. Figure S3. Rab23 enhances the migration of HSC-5 and A431 cells. Figure S4. U0126 inhibits Rab23-mediated cell migration. Table S1. Clinical characteristics of participants and protein levels of Rab23, LC3-II, Beclin1, and p62 in clinical samples. Table S2. Participant stratification according to the expression of Rab23 and autophagy proteins detected by immunohistochemical staining.

## References

[1] Janus J.M., O’Shaughnessy R.F.L., Harwood C.A., Maffucci T. (2017). Phosphoinositide 3-Kinase-Dependent Signalling Pathways in Cutaneous Squamous Cell Carcinomas. Cancers.

[2] John S.M., Trakatelli M., Gehring R., Finlay K., Fionda C., Wittlich M., Augustin M., Hilpert G., Barroso Dias J.M., Ulrich C. (2016). CONSENSUS REPORT: Recognizing non-melanoma skin cancer, including actinic keratosis, as an occupational disease—A Call to Action. J. Eur. Acad. Dermatol. Venereol..

[3] Jiang R., Fritz M., Que S.K.T. (2024). Cutaneous Squamous Cell Carcinoma: An Updated Review. Cancers.

[4] Tokez S., Wakkee M., Kan W., Venables Z.C., Mooyaart A.L., Louwman M., Nijsten T., Hollestein L.M. (2022). Cumulative incidence and disease-specific survival of metastatic cutaneous squamous cell carcinoma: A nationwide cancer registry study. J. Am. Acad. Dermatol..

[5] Colhado Rodrigues B.L., Lallo M.A., Perez E.C. (2020). The Controversial Role of Autophagy in Tumor Development: A Systematic Review. Immunol. Investig..

[6] Yun C.W., Lee S.H. (2018). The Roles of Autophagy in Cancer. Int. J. Mol. Sci..

[7] Assi M., Kimmelman A.C. (2023). Impact of context-dependent autophagy states on tumor progression. Nat. Cancer.

[8] Okura R., Nakamura M. (2011). Overexpression of autophagy-related beclin-1 in cutaneous squamous cell carcinoma with lymph-node metastasis. Eur. J. Dermatol..

[9] Yoshihara N., Takagi A., Ueno T., Ikeda S. (2014). Inverse correlation between microtubule-associated protein 1A/1B-light chain 3 and p62/sequestosome-1 expression in the progression of cutaneous squamous cell carcinoma. J. Dermatol..

[10] Choi S.R., Chung B.Y., Kim S.W., Kim C.D., Yun W.J., Lee M.W., Choi J.H., Chang S.E. (2014). Activation of autophagic pathways is related to growth inhibition and senescence in cutaneous squamous cell carcinoma. Exp. Dermatol..

[11] Zheng L.Q., Chi S.M., Li C.X. (2017). Rab23′s genetic structure, function and related diseases: A review. Biosci. Rep..

[12] Jian Q., Miao Y., Tang L., Huang M., Yang Y., Ba W., Liu Y., Chi S., Li C. (2016). Rab23 promotes squamous cell carcinoma cell migration and invasion via integrin beta1/Rac1 pathway. Oncotarget.

[13] Verburg F.A., Mader U., Luster M., Reiners C. (2018). The effects of the Union for International Cancer Control/American Joint Committee on Cancer Tumour, Node, Metastasis system version 8 on staging of differentiated thyroid cancer: A comparison to version 7. Clin. Endocrinol..

[14] Chen C., Mendez E., Houck J., Fan W., Lohavanichbutr P., Doody D., Yueh B., Futran N.D., Upton M., Farwell D.G. (2008). Gene expression profiling identifies genes predictive of oral squamous cell carcinoma. Cancer Epidemiol. Biomark. Prev..

[15] Ye H., Yu T., Temam S., Ziober B.L., Wang J., Schwartz J.L., Mao L., Wong D.T., Zhou X. (2008). Transcriptomic dissection of tongue squamous cell carcinoma. BMC Genom..

[16] Padilla R.S., Sebastian S., Jiang Z., Nindl I., Larson R. (2010). Gene expression patterns of normal human skin, actinic keratosis, and squamous cell carcinoma: A spectrum of disease progression. Arch. Dermatol..

[17] Nindl I., Dang C., Forschner T., Kuban R.J., Meyer T., Sterry W., Stockfleth E. (2006). Identification of differentially expressed genes in cutaneous squamous cell carcinoma by microarray expression profiling. Mol. Cancer.

[18] Warren T.A., Broit N., Simmons J.L., Pierce C.J., Chawla S., Lambie D.L., Quagliotto G., Brown I.S., Parsons P.G., Panizza B.J. (2016). Expression profiling of cutaneous squamous cell carcinoma with perineural invasion implicates the p53 pathway in the process. Sci. Rep..

[19] Cerami E., Gao J., Dogrusoz U., Gross B.E., Sumer S.O., Aksoy B.A., Jacobsen A., Byrne C.J., Heuer M.L., Larsson E. (2012). The cBio cancer genomics portal: An open platform for exploring multidimensional cancer genomics data. Cancer Discov..

[20] Charafe-Jauffret E., Tarpin C., Bardou V.J., Bertucci F., Ginestier C., Braud A.C., Puig B., Geneix J., Hassoun J., Birnbaum D. (2004). Immunophenotypic analysis of inflammatory breast cancers: Identification of an ‘inflammatory signature’. J. Pathol..

[21] Biederbick A., Kern H.F., Elsasser H.P. (1995). Monodansylcadaverine (MDC) is a specific in vivo marker for autophagic vacuoles. Eur. J. Cell Biol..

[22] Haga K., Fukuda M. (2025). Comprehensive knockout analysis of the RAB family small GTPases reveals an overlapping role of RAB2 and RAB14 in autophagosome maturation. Autophagy.

[23] Kang S., Liu S., Dong X., Li H., Qian Y., Dai A., He W., Li X., Chen Q., Wang H. (2025). USP4 depletion-driven RAB7A ubiquitylation impairs autophagosome-lysosome fusion and aggravates periodontitis. Autophagy.

[24] Zhao Y.G. (2025). Rab GTPases as “eat me” signals for selective autophagy. J. Cell Biol..

[25] Sharifi M.N., Mowers E.E., Drake L.E., Collier C., Chen H., Zamora M., Mui S., Macleod K.F. (2016). Autophagy Promotes Focal Adhesion Disassembly and Cell Motility of Metastatic Tumor Cells through the Direct Interaction of Paxillin with LC3. Cell Rep..

[26] Han Q., Deng Y., Chen S., Chen R., Yang M., Zhang Z., Sun X., Wang W., He Y., Wang F. (2017). Downregulation of ATG5-dependent macroautophagy by chaperone-mediated autophagy promotes breast cancer cell metastasis. Sci. Rep..

[27] Xia H., Green D.R., Zou W. (2021). Autophagy in tumour immunity and therapy. Nat. Rev. Cancer.

[28] Russell R.C., Guan K.L. (2022). The multifaceted role of autophagy in cancer. Embo J..

[29] Zhou Z., Liu J., Fu T., Wu P., Peng C., Gong X., Wang Y., Zhang M., Li Y., Wang Y. (2021). Phosphorylation regulates the binding of autophagy receptors to FIP200 Claw domain for selective autophagy initiation. Nat. Commun..

[30] Zou C.G., Ma Y.C., Dai L.L., Zhang K.Q. (2014). Autophagy protects C. elegans against necrosis during Pseudomonas aeruginosa infection. Proc. Natl. Acad. Sci. USA.

[31] Wright T.J., McKee C., Birch-Machin M.A., Ellis R., Armstrong J.L., Lovat P.E. (2013). Increasing the therapeutic efficacy of docetaxel for cutaneous squamous cell carcinoma through the combined inhibition of phosphatidylinositol 3-kinase/AKT signalling and autophagy. Clin. Exp. Dermatol..

[32] Claerhout S., Verschooten L., Van Kelst S., De Vos R., Proby C., Agostinis P., Garmyn M. (2010). Concomitant inhibition of AKT and autophagy is required for efficient cisplatin-induced apoptosis of metastatic skin carcinoma. Int. J. Cancer.

[33] Stalnecker C.A., Grover K.R., Edwards A.C., Coleman M.F., Yang R., DeLiberty J.M., Papke B., Goodwin C.M., Pierobon M., Petricoin E.F. (2022). Concurrent Inhibition of IGF1R and ERK Increases Pancreatic Cancer Sensitivity to Autophagy Inhibitors. Cancer Res..

[34] You B., Xia T., Gu M., Zhang Z., Zhang Q., Shen J., Fan Y., Yao H., Pan S., Lu Y. (2022). AMPK-mTOR-Mediated Activation of Autophagy Promotes Formation of Dormant Polyploid Giant Cancer Cells. Cancer Res..

[35] Debnath J., Gammoh N., Ryan K.M. (2023). Autophagy and autophagy-related pathways in cancer. Nat. Rev. Mol. Cell Biol..

