# Supplementary material for: Rab23 Drives Cutaneous Squamous Cell Carcinoma Invasion via ERK-Mediated Autophagy Activation
Source: Cancers (Basel). 2026 Oct 26;18(21):3458. doi: 10.3390/cancers18213458 (PMC13761739; doi:10.3390/cancers18213458)
Supplement: Supplementary file 1 [file cancers-18-03458-s001.zip › cancers-4527201-supplementary.pdf]

Rab23 Drives Cutaneous Squamous Cell Carcinoma Invasion via ERK-Mediated Autophagy Activation

*Jiaqiang Wu[1†], Ziye Feng[1†], Chunyu Xie[2], Jingrun Yang[1]**

[1]Department of General Surgery, the First Medical Center of Chinese PLA General Hospital, Beijing, China.

[2]CAS Key Laboratory for Biomedical Effects of Nanomaterials and Nanosafety, CAS Center for Excellence in Nanoscience, National Center for Nanoscience and Technology, Chinese Academy of Sciences, Beijing, China.

[†]These authors have contributed equally to this work and share first authorship.

***Corresponding author:**

Jingrun Yang

Department of General Surgery, The First Medical Center of Chinese PLA General Hospital

No. 28 Fuxing Road, Hai Dian District, Beijing, 100853, China

E-mail: yangjingrun@301hospital.com.cn

Supplementary Figures 1-4
Supplementary Tables 1-2

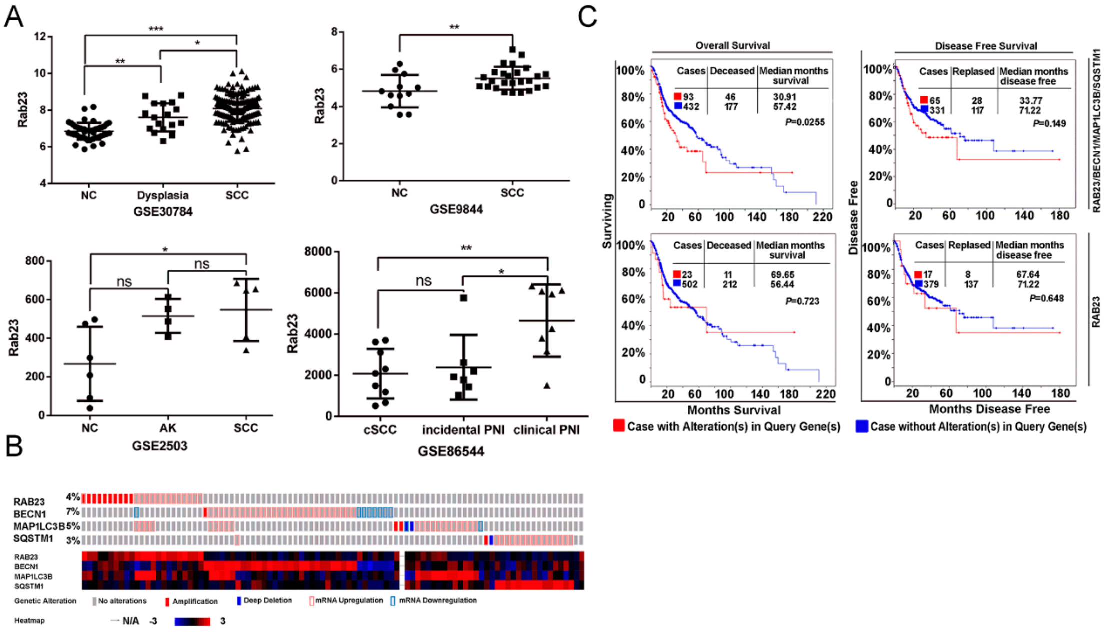

Supplementary Figure 1. Rab23 mRNA was overexpressed in patients with cSCC from GEO and TCGA datasets and correlated with shorter overall survival. (**A**) Upregulation of Rab23 mRNA expression was observed in cSCC patients from different GEO datasets. (**B**) Oncoprint of mRNA expression of Rab23, Beclin1, LC3-II, and p62 in a TCGA dataset containing 528 cases with cSCC. (**C**) Kaplan–Meier curves were generated to analyze the correlation of Rab23 alone or Rab23/Beclin1/LC3-II/p62 in combination with the overall survival or disease-free survival of patients in the TCGA dataset. The blue line represents the low-expression group; the red line represents the high-expression group. *$P < 0.05$, **$P < 0.01$, ***$P < 0.001$. NC: negative control; BECN1: Beclin1.

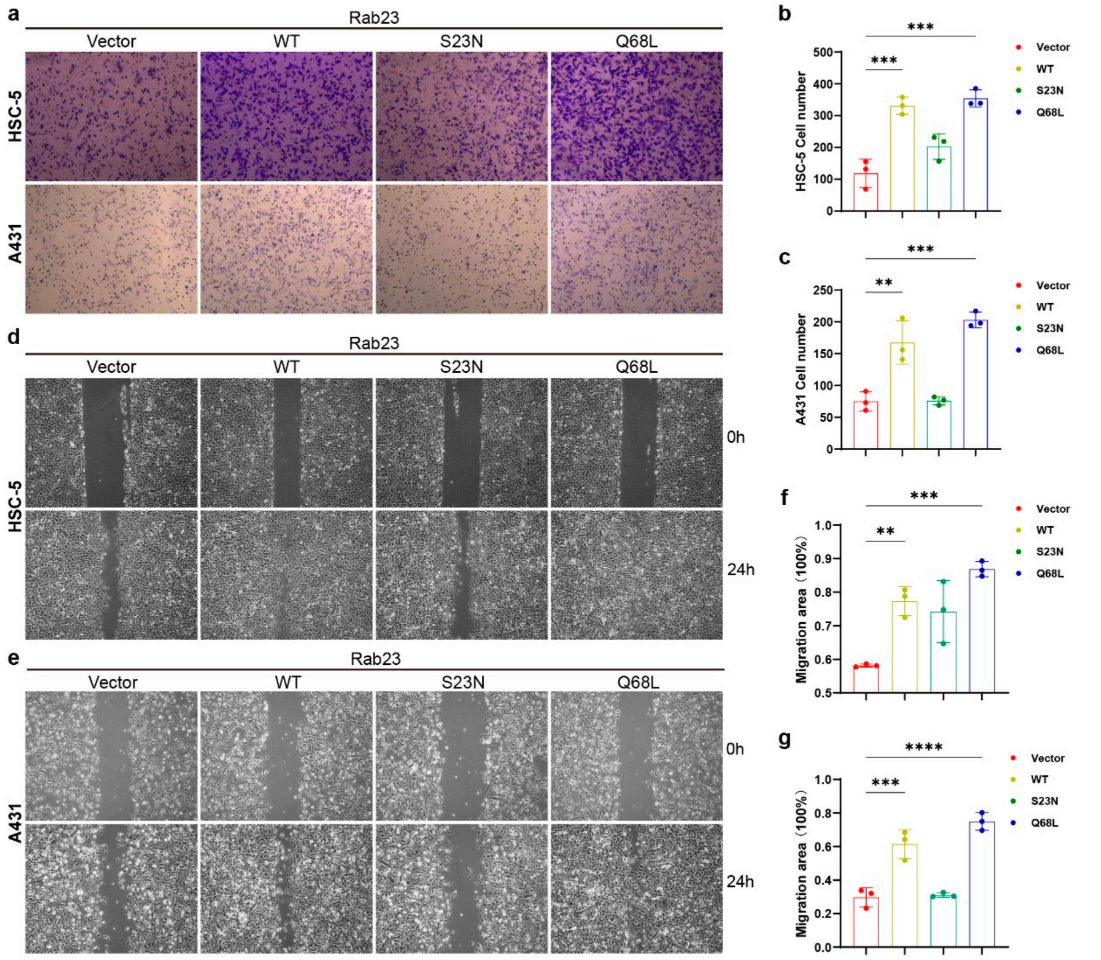

Supplementary Figure 2. Rab23 enhances the migration of HSC-5 and A431 cells. (a–c) Transwell assays showing the migratory ability of HSC-5 and A431 cells transfected with Vector, Rab23 WT, S23N, or Q68L. Quantification of migrated cells is shown in b and c. (d–g) Wound-healing assays showing cell migration at 0 h and 24 h after transfection. Migration areas were quantified for HSC-5 cells (f) and A431 cells (g). Data are presented as mean ± SD, n = 3. **$P < 0.01$, ***$P < 0.001$, ****$P < 0.0001$.

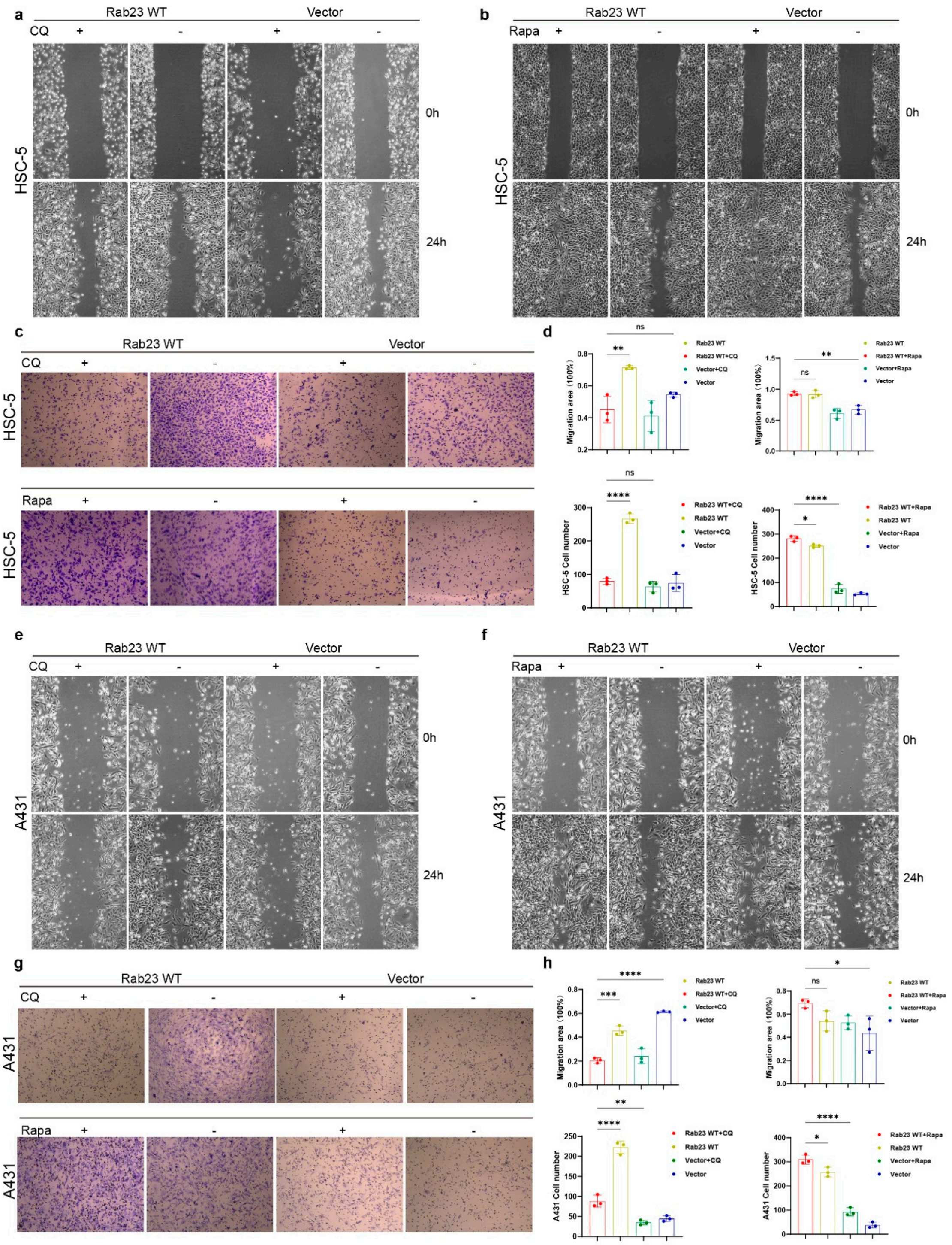

Supplementary Figure 3. Rab23 enhances the migration of HSC-5 and A431 cells. Autophagy regulates Rab23-mediated cell migration. (a–d) Wound-healing and Transwell assays showing the effects of chloroquine (CQ) or rapamycin (Rapa) on Rab23 WT-mediated migration in HSC-5 cells. Quantification of migration area and migrated cell number is shown in d. (e–h) Wound-healing and Transwell assays showing the effects of CQ or Rapa on Rab23 WT-mediated migration in A431 cells. Quantification is shown in h. Data are presented as mean ± SD, n = 3. ns, not significant; *P < 0.05, **P < 0.01, ***P < 0.001, ****P < 0.0001.

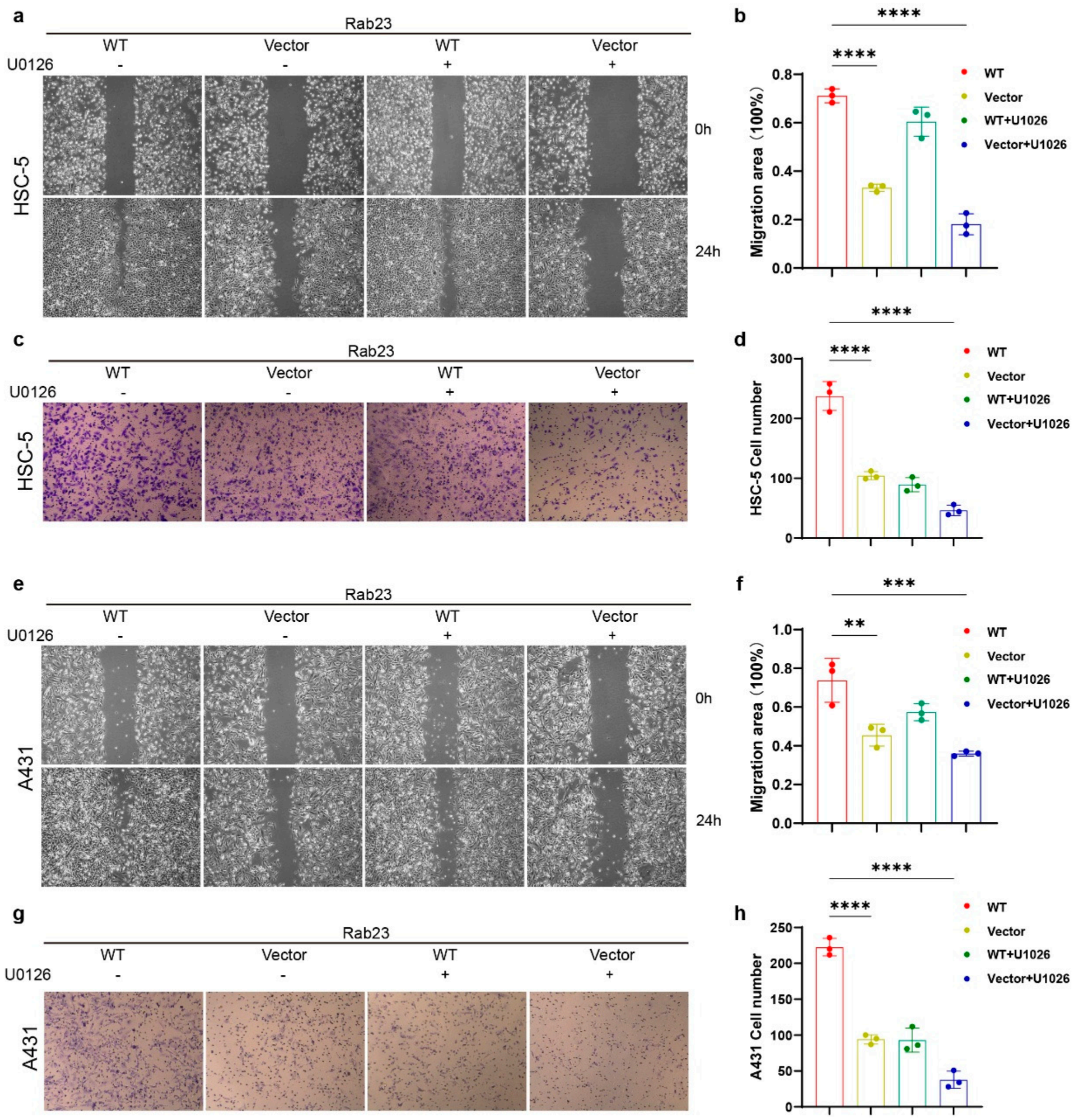

Supplementary Figure 4. U0126 inhibits Rab23-mediated cell migration. (a-d) Wound-healing and Transwell assays showing the effect of U0126 on Rab23 WT-mediated migration in HSC-5 cells. Quantification of migration area and migrated cell number is shown in b and d. (e–h) Wound-healing and Transwell assays showing the effect of U0126 on Rab23 WT-mediated migration in A431 cells. Quantification is shown in f and h. Data are presented as mean ± SD, n = 3. **P < 0.01, ***P < 0.001, ****P < 0.0001.

Table S1. Clinical characteristics of participants and protein levels of Rab23, LC3-II, Beclin1, and p62 in clinical samples.

Clinicopathological features	n	Rab23		*P*	Beclin1		*P*	LC3B		*P*	P62		*P*
		Low	High		Low	High		Low	High		Low	High	
Disease duration													
<1year	11	6	5		7	4		6	5		9	2	
				0.127[b]			0.235[b]			0.383[b]			1.000[b]
≥1year	30	7	23		11	19		10	20		26	4	
Age													
<65years	18	8	10		8	10		7	11		15	3	
				0.121			0.951			0.987			1.000[b]
≥65years	23	5	18		10	13		9	14		20	3	
Sex													
Male	18	6	12		8	10		6	12		14	4	
				0.843			0.951			0.509			0.441[b]
Female	23	7	16		10	13		10	13		21	2	
Tumor size													
<2cm	14	7	7		10	4		8	6		12	2	
				0.145[b]			**0.011**			0.087			1.000[b]
≥2cm	27	6	21		8	19		8	19		23	4	
Predominant differentiation													
Well	26	12	14	**0.023[b]**	16	10	**0.003**	14	12	**0.010[b]**	25	1	**0.034[b]**

M/ P	15	1	14		2	13		2	13		10	5	
UICC stage													
I/II	32	13	20		18	14		16	16		28	4	
				0.231[b]			**0.009[b]**			**0.020b**			0.845[b]
III/IV	9	1	8		0	9		0	9		7	2	
Lymphovascular invasion													
Absent	34	12	22		16	18		14	20		31	3	
				0.523[b]			0.632			0.844			0.083[b]
Present	7	1	6		2	5		2	5		4	3	
Lymph node metastasis													
Absent	36	13	23		18	18		15	21		31	5	
				0.266[b]			0.103[b]			0.659[b]			1.000[b]
Present	5	0	5		0	5		1	4		4	1	
Perineural Invasion													
Absent	35	8	27		15	20		13	22		31	4	
				0.129[b]			1.000[b]			0.886[b]			0.437
Present	6	5	1		3	3		3	3		4	2	

Table S2. Participant stratification according to the expression of Rab23 and autophagy proteins detected by immunohistochemical staining.

Group	n	Rab23		*p*	Beclin1		*p*	LC3B		*p*	P62		*p*
		Low	High		Low	High		Low	High		Low	High	
Normal Skin	30	30	0	0.000	25	5	0.001	23	7	0.004	15	15	0.001
AK	49	20	29		23	26		22	27		26	23	
cSCC	41	13	28		18	23		16	25		35	6	
